# Supplementary material for: Single Amino Acid Substitutions in the Cucumber Mosaic Virus 1a Protein Induce Necrotic Cell Death in Virus-Inoculated Leaves without Affecting Virus Multiplication
Source: Viruses. 2020 Jan 13;12(1):91. doi: 10.3390/v12010091 (PMC7019621; doi:10.3390/v12010091)
Supplement: Supplementary file 1 [file viruses-12-00091-s001.zip › Tian et al. Figure S1-S10.pdf]

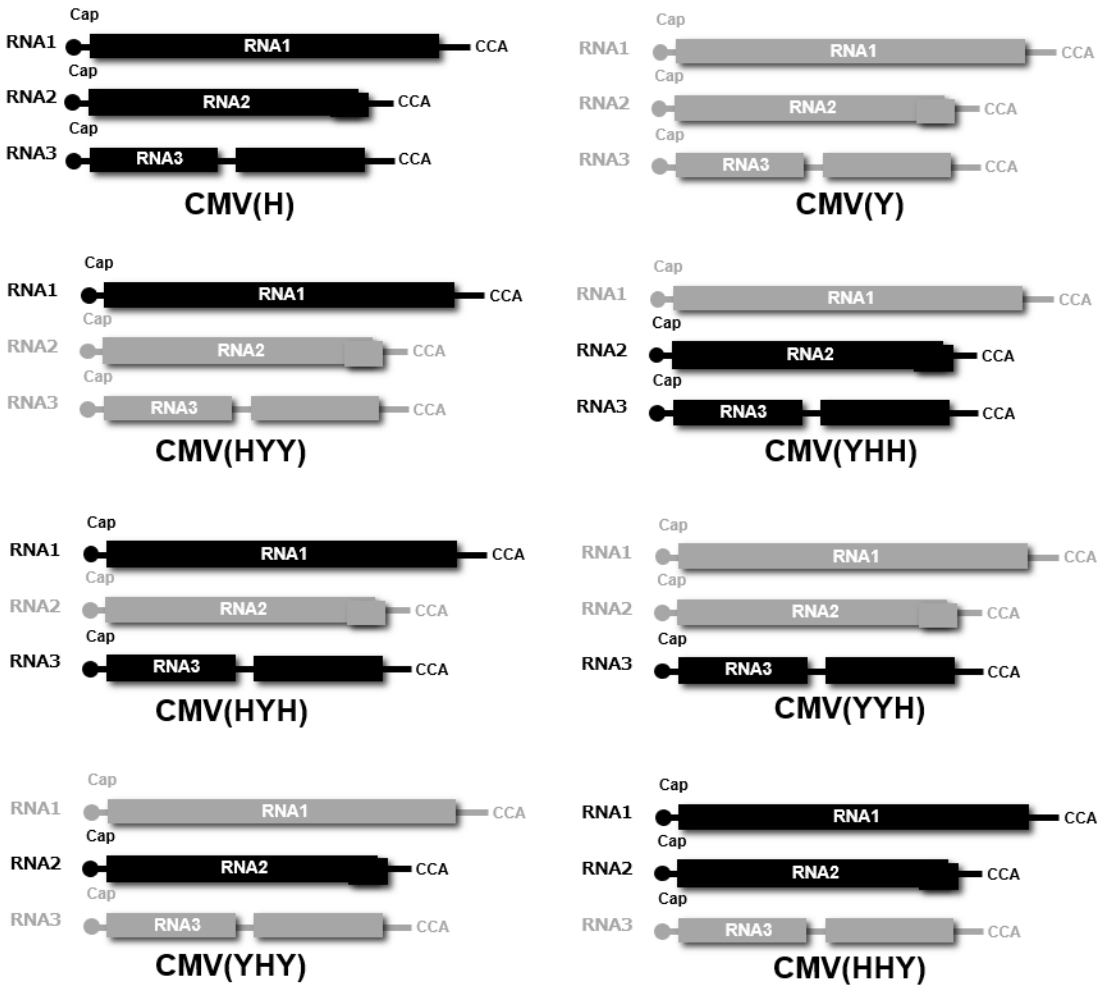

Figure S1. Schematic diagrams of the RNA genomes of the reassortant CMVs, derived from RNA1, RNA2, and RNA3 of CMV(H) and CMV(Y) (upper part of figure), used in the present study. Black indicates the CMV(H) genomes; gray, the CMV(Y) genomes.

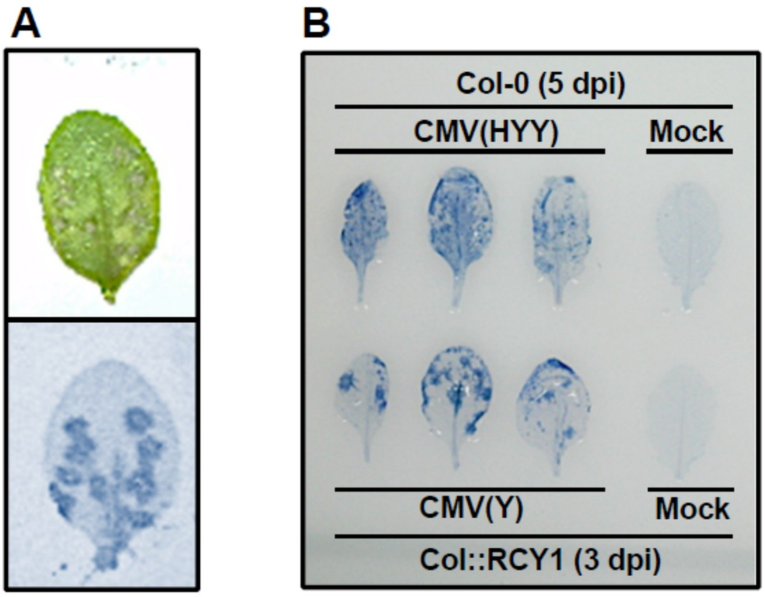

Figure S2. Visualization of cell death in CMV(HYY)- or CMV(Y)-inoculated Arabidopsis thaliana Col-0 leaves. (A) HR cell death in CMV(Y)-inoculated A. thaliana Col::RCY1 at 3 days post-inoculation (dpi) under bright field (upper panel), and with trypan blue staining (lower panel). (B) Cell death visualized by trypan blue staining: three independent CMV(HYY)-inoculated A. thaliana Col-0 leaves at 5 dpi; HR cell death in three independent CMV(Y)-inoculated A. thaliana Col::RCY1 leaves at 3 dpi; and mock-inoculated A. thaliana Col-0 leaves at 3 and 5 days.

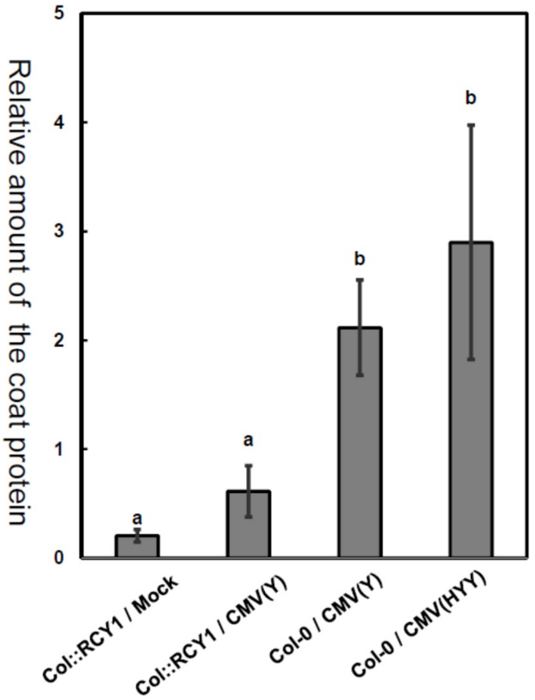

Figure S3. Accumulation of CMV coat protein in CMV(HYY)- or CMV(Y)-inoculated Arabidopsis thaliana Col-0 leaves at 5 days after inoculation. Coat protein (CP) was quantitatively measured using ELISA. Vertical error bars indicate standard error of the mean for CP quantity from three independent samples; bars with the same letter indicate no significant difference between these treatments at $p < 0.05$ by LSD test.

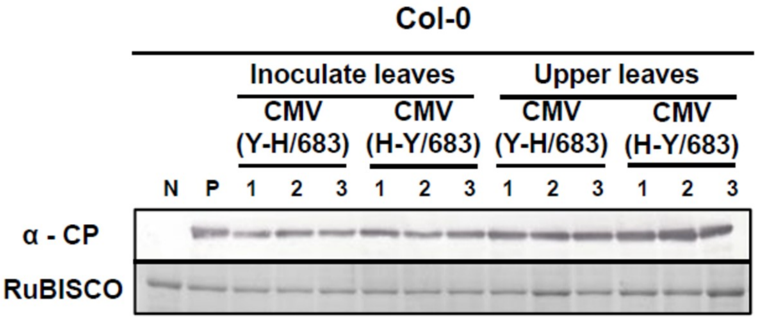

Figure S4. Detection of CMV coat protein in extracts of virus-inoculated leaves and non-inoculated upper Arabidopsis thaliana Col-0 leaves. Extracts from fully expanded leaves of three independent Col-0 plants (numbers 1, 2, and 3) inoculated with reassortant CMV: CMV(H-I/683) and CMV(Y-I/683) were analyzed at 7 dpi using western blotting, as also were extracts from non-inoculated upper leaves of corresponding plants. RuBISCO protein is shown as an internal reference for protein quantity. CMV coat protein in CMV(Y)-inoculated leaves was quantified as a positive (P) control, and mock-inoculated leaves as a negative (N) control.

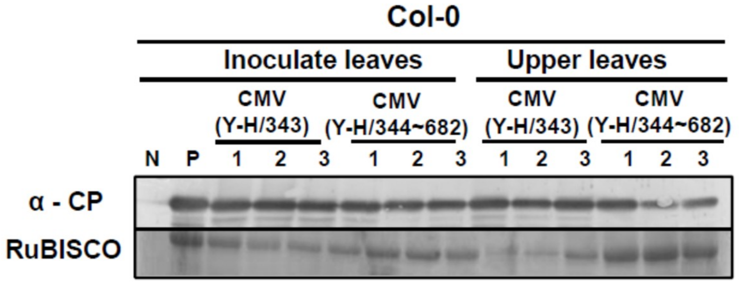

Figure S5. Detection of CMV coat protein in extracts of virus-inoculated leaves and non-inoculated upper Arabidopsis thaliana Col-0 leaves. Fully expanded leaves of three independent Col-0 plants (number 1, 2, and 3), which were inoculated with reassortants CMV(Y-H/343) or CMV(Y-H/344~682), were analyzed at 7 dpi by western blotting, as also were extracts from non-inoculated upper leaves of the corresponding plants. RuBISCO protein is shown as an internal reference for protein quantity. Positive (P) and negative (N) controls quantified as explained in Fig. S4 legend.

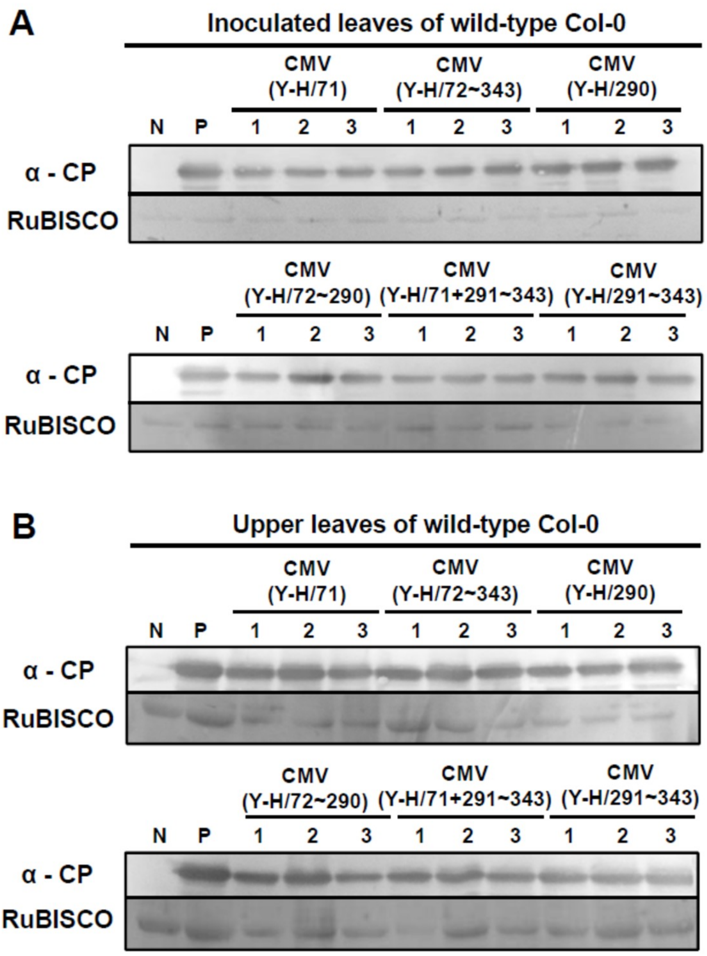

Figure S6. Detection of CMV coat protein in extracts of virus-inoculated Arabidopsis thaliana Col-0 leaves and non-inoculated upper leaves. (A) Fully expanded leaves of three independent Col-0 plants (numbers 1, 2, and 3) inoculated with reassortant CMVs. CMV(Y-H/71), CMV(Y-H/72~343), CMV(Y-H/290), CMV(Y-H/72~290), CMV(Y-H/71+291~343), and CMV(Y-H/291~343) were analyzed at 7 dpi by western blotting. (B) Non-inoculated upper leaves of the corresponding plants were analyzed at 7 dpi by western blotting. RuBISCO protein is shown as an internal reference for protein quantity. Positive (P) and negative (N) controls quantified as explained in Fig. S4 legend.

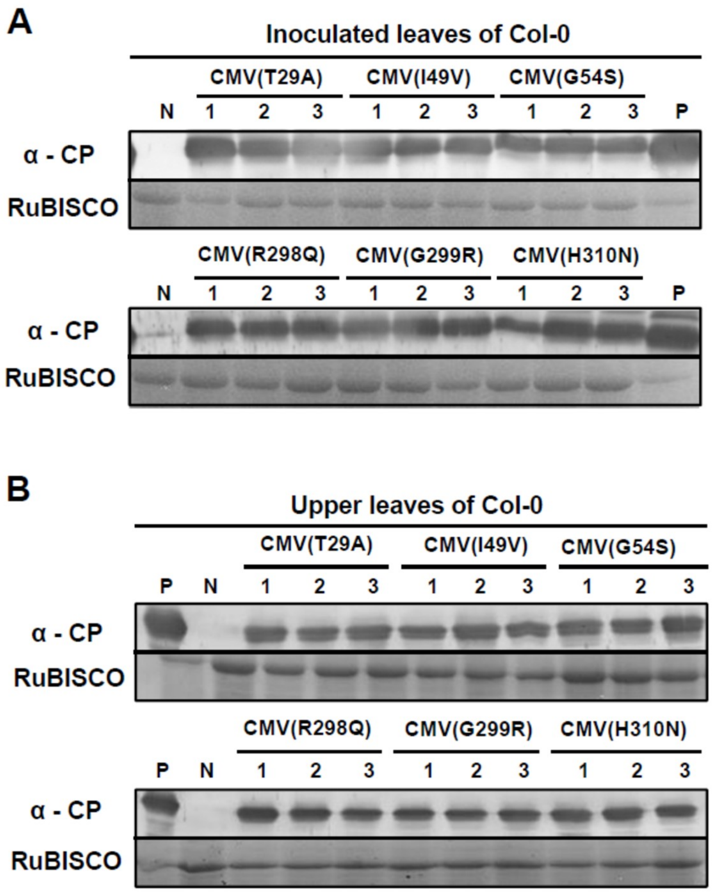

Figure S7. Detection of CMV coat protein in extracts of virus-inoculated Arabidopsis thaliana Col-0 leaves and non-inoculated upper leaves. (A) Fully expanded leaves of three independent Col-0 plants (number 1, 2, and 3) inoculated with one of six CMVs with different single amino-acid substitutions were analyzed at 7 days post-inoculation (dpi) by western blotting. (B) Non-inoculated upper leaves of the corresponding plants were analyzed at 7 dpi by western blotting. RuBISCO protein is shown as an internal reference for protein quantity. Positive (P) and negative (N) controls quantified as explained in Fig. S4 legend.

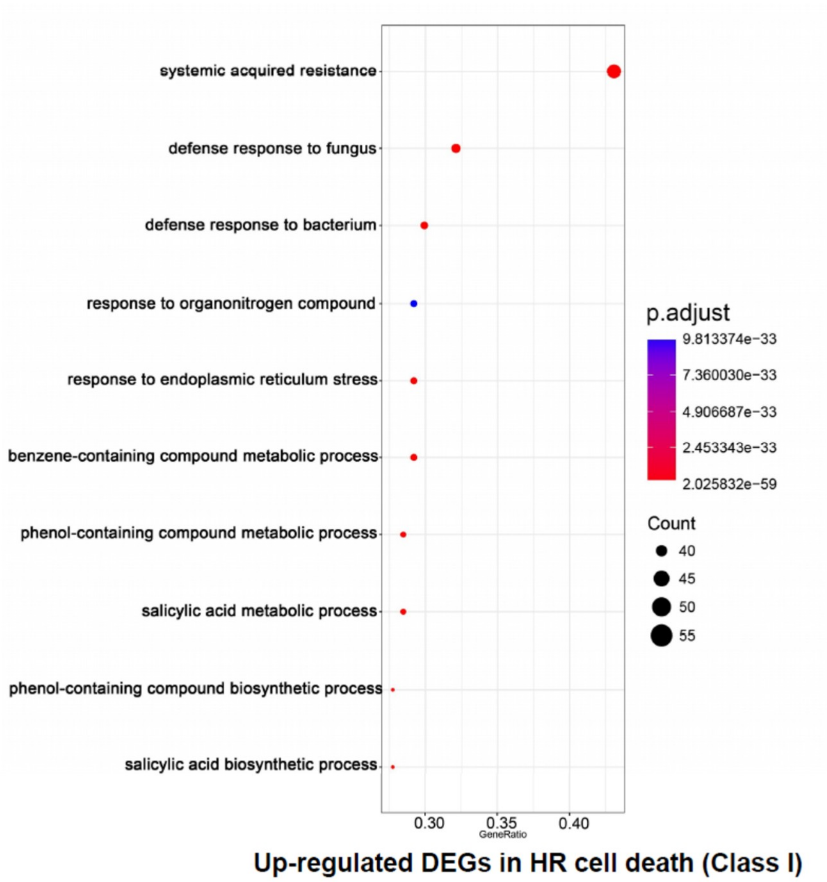

Figure S8. Gene ontology enrichment analysis for 149 up-regulated differentially-expressed genes specific in CMV(Y)-inoculated Col::RCY1 leaves showing HR cell death. The top 10 GO enrichment terms in BP for the DEGs are shown. Dot size is proportional to gene number (Count); dot color represents p.adjust value.

Tian et al. Figure S9

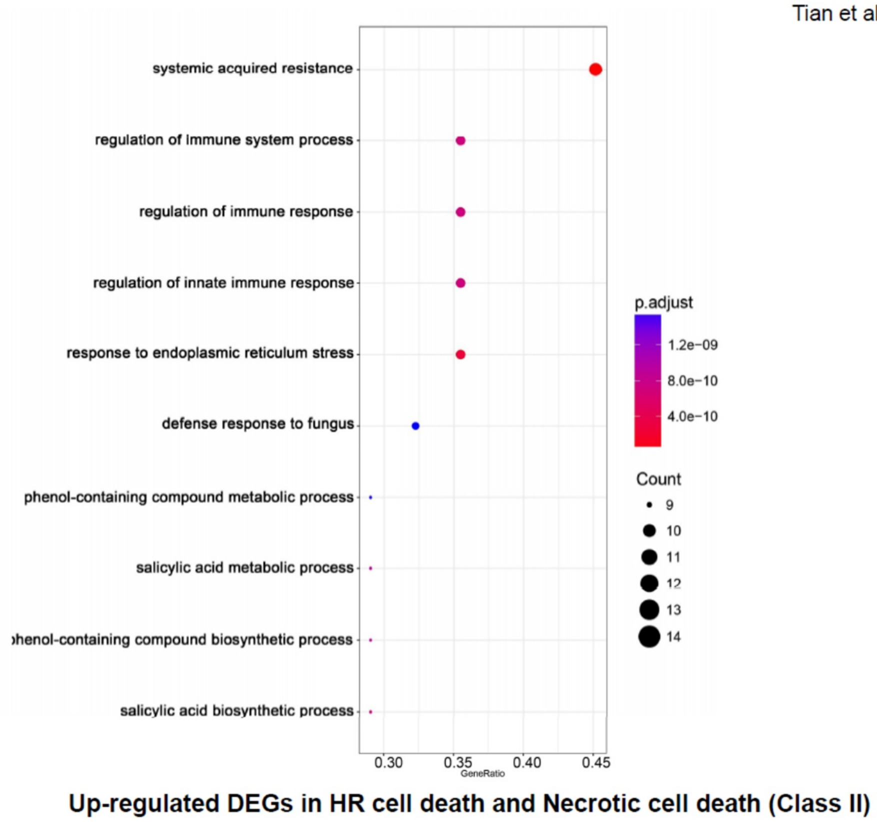

Figure S9. Gene ontology enrichment analysis for 35 up-regulated genes in both CMV(HYY)-inoculated Col-0 leaves showing necrotic cell death and CMV(Y)-inoculated Col-0::RCY1 leaves showing HR cell death. The top 10 GO enrichment terms in BP for the DEGs are shown. Dot size and color as in Fig. S8.

Tian et al. Figure S10

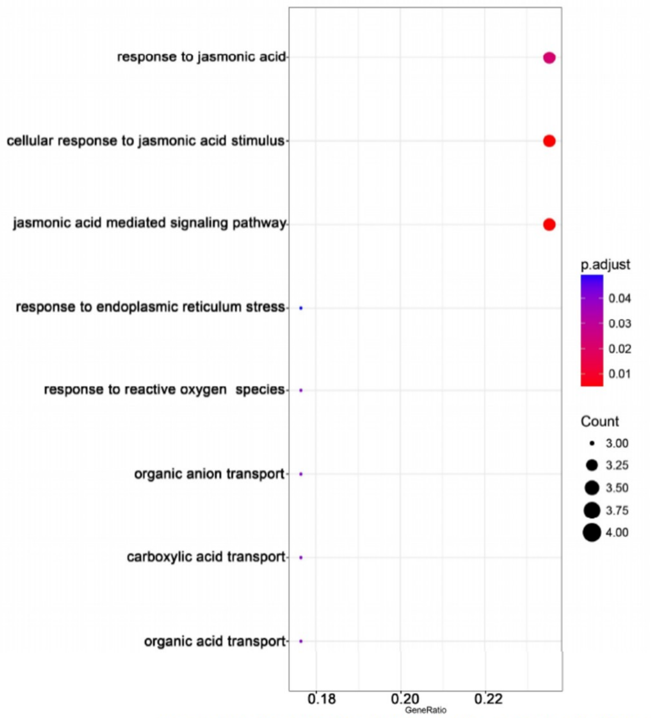

Up-regulated DEGs in Necrotic cell death (Class III)

Figure S10. Gene ontology enrichment analysis for 17 up-regulated genes specific in CMV(HYY)-inoculated Col-0 leaves showing necrotic cell death. The GO enrichment terms in BP for the DEGs are shown. Dot size and color as in Fig. S8.
